# Improving the Genetics of Tuber Yield and Resistance to Mite to Avoid Mite Incident and to Increase the Productivity of Cassava (*Manihot esculenta* Crantz)

**DOI:** 10.1155/2022/6309679

**Published:** 2022-06-16

**Authors:** Sholihin, Sri W. Indiati, Kartika Noerwijati, Tinuk S. Wahyuni, Heru Kuswantoro, Suhartina, Rudy Soehendi, Marida S. Y. I. Bayu, Gatut W. A. Sutanto, Made J. Mejaya, Rohmad Budiono

**Affiliations:** ^1^Indonesian Legumes and Tuber Crops Research Institute, Jl. Raya Kendalpayak, Km 8, P.O. Box 66, Malang 65101, East Java, Indonesia; ^**2**^ Assessment Institute for Agricultural Technology East Java, Indonesian Agency for Agriculture Research and Development, Ministry of Agriculture, Malang, Indonesia

## Abstract

Mite (*Tetranychus urticae)* that attacks the cassava plants during dry season can reduce the yield up to 53%, depending on plant age and duration of attacks. The objective of the trial was to evaluate the cassava promising clones for tuber root yield and mite resistance. The field trial was done in Malang, East Java, Indonesia, in 2018 with fifteen clones using a randomized complete block design, with three replications. The glass house experiment for mite evaluation was done in Malang in 2018. A total of fifteen clones were used in this glass house trial. Infestation of mite (imago) was done 1 month after plating with 15 mites/pot on the lower part of the fourth or fifth leaf. Results of the field experiment showed that there was a genetic variability in the clones tested. The fresh tuber yield in 10 months ranged 30.33–55.67 t/ha with mean 41.34 t/ha. The fresh tuber yield of clone OMM 0915-11 was the highest of 55.67 t/ha. The tuber result of clone UJ5d50-207-3 similar to OMM 0915-11 and significantly higher than check variety UJ5. Response of clones to mite attack were as follows: two clones were resistant, ten clones were moderately resistant, and the other clones were susceptible. Based on the green house trial, the response of clones to mite attack was as follows: one clone was highly resistant, two clones were resistant, ten clones were moderately resistant, and the other clones were susceptible. Clone OMM 0915-11 was resistant variety or high resistant variety based on the green house and field experiments, while clone UJ5d50-207-3 was moderately resistant based on both glass house and field experiments.

## 1. Introduction

Productivity of cassava is important characteristic related to farmer income per crop area and harvesting time. Productivity of cassava is determined by genetic factor and environment factor. Many released varieties can be used by farmers. Air temperature, sun light, and rain fall are the environment factors that can not be modified, while fertilizer (dosage and kind), planting distance, and irrigation are the environment factors that can be modified.

Fertilization is one of the environmental factors that can be modified to increase productivity of cassava. Kind and dosage of fertilizer needed to get the high productivity depended on the soil fertility and the kind of variety used. Senkero et al. [1] reported that fertilization 0–80 kg·N/ha gave the significantly positive response to yield in all locations of the experiments with characteristic as follows: N content in the soil 0.5–1.4 g/kg, K 0.15–0.81 cmolc/kg, and P 7.6–16 mg/kg, while fertilization with 0–22.5 kg·P/ha and dan K 0-30 kg/ha did not give significant response to yield.

The cassava productivity is also affected by insect severity that attacks the crops in the field. An important insect in cassava is mite (*Tetranychus urticae*) [2]. In general, this insect attacks the plant during dry season. Mite can reduce the yield 20–53%, depending on plant age and duration of attacks. Indiati [3] reported the loss of yield of 8–54%. Ezenwaka et al. [4] reported that the loss of yield of cassava due to green mite (*Mononychellus tanajoa* (Bondar)) was over 80%. To handle the mite attack, the farmer uses resistance variety. Use of resistance variety is easy and cheap and causes no pollution for the environment. So, new varieties should be moderately resistant or resistant to mite.

The role of plant breeding is important in increasing the productivity and the mite resistance of cassava variety. Conventional breeding is still dominant in producing new varieties. Germplasm evaluation is first step in conventional breeding, followed by clonal selection. Clonal selection involves single plant selection, single row selection, and single plot selection. After that, they are followed by preliminary yield trial and advanced yield trial [5].

There were some promising clones from breeding activity that started in 2009. Some promising clones resulted from hybridization and some others resulted from mutation using gamma irradiations. The objective of the trial was to evaluate the cassava promising clones for tuber root yield and to determine the mite resistance.

## 2. Materials and Methods

### 2.1. Study Sites

The study consisted of field and glass house experiments which were conducted in Malang, East Java, Indonesia, in 2018. The field experiment was conducted in the Jambegede Experiment Farm which is located at the coordinate of −8° 10′20″ South Latitude and 112° 33′43″ East Longitude, with 335 m above sea level. The glass house experiment was conducted in Kendalpayak which is located at the coordinate of −8°2′51″ South Latitude and 112°37′30″ East Longitude, with the altitude of 436 m above sea level.

### 2.2. Plant Materials

#### 2.2.1. Field Experiment

The field experiment was done using a randomized complete block design, with three replications. A total of fifteen clones were tested in this experiment. The plot size was a 5 m × 4.8 m and plant distance was a 100 cm × 80 cm. Plants were fertilized three times, first fertilization was done in 2 weeks after planting 15 grams of *phonska* (N 15%, P 15%, K 15%)/plants, second fertilization was done in six weeks after planting 20 grams of *phonska* (N 15%, P 15%, K 15%)/plant, and third fertilization was done in 10 weeks after planting 20 grams of *phonska* (N 15%, P 15%, K 15%)/plant.

#### 2.2.2. Glass House Experiment

The glass house experiment for mite evaluation was done using a randomized complete block design, with three replications. A total of fifteen clones, CId50-223, OMM0916-2, UJ5d50-11, OMM 0915-11, CM 7514-7, SM 1219-9, CM 4867-1, UJ3d30-553-1, UJ3d30-511-2, UJ5d50-99-1, dan UJ5d50-207-3, UJ3, UJ5, Adira 4, and Litbang UK2, were used in this trial.

Cassava stake with a 25 cm long was planted in a pot (1 plant/pot). Diameter of pot was 30 cm with 5 kg soil/pot. Fertilization was 5 grams of *phonska* (N 15%, P 15%, K 15. Irrigation was given based on the needs. Infestation of mite (imago) was done in 1 month after planting with 15 mites/pot on the lower part of the fourth or fifth leaf.

### 2.3. Data Collection

#### 2.3.1. Field Experiment

The variables taken were the fresh tuber yield, starch content, and plant height in seven months, number of tubers/plant, diameter of tuber, harvest index, and mite attack with natural infestation. Starch content was taken based on gravity system with wet basis. Starch content was obtained from multiplication between specific gravity and 112.1 minus 106.4. Specific gravity was gotten from the fresh tuber weight in the air divided by the value of the fresh tuber in the air minus the fresh tuber in the water.

The damage intensity of the mite attacks was calculated as follows:

(1) $$\begin{array}{c} I=\sum \frac{nxv}{NxV}\times 100\%, \end{array}$$

where *I* is the damage intensity of the mite attack, *N* is the number of leaves/plant, *V* is the highest score (5), *n*is the number of leaves in each score category, and *v* is category score (from 0 to 5).

Scoring was done as follows: 0 = no symptoms; 1 = Initiation of yellowish spots on some of the lower and/or middle leaves; 2 = fairly abundant yellowish spots on lower and/or middle leaves; 3 = considerable damage: many spots; small necrotic zones and curling of leaves, especially the basal and middle leaves; yellowing and loss of some leaves; 4 = severe damage: heavy defoliation in the lower and middle part of the plant; a large number of mites as well as webs can be observed; 5 = total defoliation of the plant; shoot reduced in size with large number of webs; death of plant.

Susceptibility class was determined using the Chiang and Talekar [6] formulas as follows:

(2) $$\begin{array}{c} \text{Highly resistant}\left(\text{HR}\right)=I<\left(\bar{I}-2\delta \right),\\ \text{Resistant}\left(R\right)=\left(\bar{I}-2\delta \right)<I<\left(\bar{I}-\delta \right),\\ \text{Moderately Resistant}\left(\text{MR}\right)=\left(\bar{I}-\delta \right)<I<\left(\bar{I}+\delta \right),\\ \text{Susceptible}\left(S\right)=I>\left(\bar{I}+\delta \right), \end{array}$$

where $\left(\bar{I}\right)$ is mean of the damage intensity of the mite attack. *δ* is standard deviation.

#### 2.3.2. Glass House Experiment

Data were taken on mite population during 3–9 weeks after planting, the damage intensity of mite attack during 3–9 weeks after planting, color of leaf in 3 months after planting, and pubescent of leaf in 3 months after planting.

### 2.4. Data Analysis

The damage intensity of the mite attacks and susceptibility class was calculated using a MSTAT-C software to analyze the data and Minitab software was used to develop the dendogram.

## 3. Results

### 3.1. The Field Experiments

#### 3.1.1. Agronomic Traits of Cassava Promising Clones

The plant height character contributes to the yield. Plant height of tested clones was 119–157 cm with average 134 cm. Plant height of clone CM 3306-19 was the lowest, while clone UJ3d30-553-1 was the highest. The fresh tuber yield in 10 months ranged between 30.33 and 55.67 t/ha with mean 41.34 t/ha (Table 1). The fresh tuber yield of OMM 0915-11 was the highest, 55.67 t/ha. The tuber result of SM 1219-9, UJ5d50-207-3, Litbang UK2, and CM 3306-19 was similar to OMM 0915-11. Starch content in 10 months of tested clones was in the range of 16.83–21.0%, with mean 19.313%. Clone CM 4867-1 had the highest starch content (21%). Clones CM 3306-19, OMM0916-2, OMM 0940-2, CM 7514-7, SM 1219-9, CM 4867-1, UJ3, UJ5, Adira 4, Litbang UK2, UJ3d30-553-1, UJ5d50-99-1, and UJ5d50-207-3 had starch content similar to CM 4867-1. Starch yield in 10 months of tested clones ranged between 5.87 and 10.89 t/ha with average 7.97 t/ha. The highest starch yield (10.89 t/ha) was achieved by clone UJ5d50-207-3. Clones UJ5d50-207-3, SM 1219-9, OMM 0915-11, CM 3306-19, Litbang UK2, and UJ5d50-99-1 had starch yield similar to UJ5d50-207-3.

Mite attacks intensity in 10 months of plant age ranged between 24.14% and 51.32% (Table 2). Clone OMM 0915-11 had the lowest intensity of mite attacks (24.14%), which was resistant variety. Clone OMM 0940-2 was also resistant variety. Clones UJ5d50-99-1 and UJ5d50-207-3 were moderately resistant variety, while UJ5 was susceptible variety (Table 2). Harvest index of tested clones was in the range of 0.48–0.65. Clone CM 3306-19 had the highest harvest index (0.65). Clone UJ5d50-99-1 had harvest index similar to CM 3306-19 and significantly higher than UJ5. The average tuber (≤100 gram) diameter ranged from 4.36 to 5.87 cm. Variety UJ5 had the biggest diameter (5.87 cm). The tuber of clone OMM 0915-11 was similar to UJ5. The number of tubers (≤100 gram) ranged from 9 to 17 tubers/plant; however, this difference was not significant statistically. Tuber length of tested clones ranged from 20 to 29 cm; however, this difference was not significant statistically.

**Figure 1:**
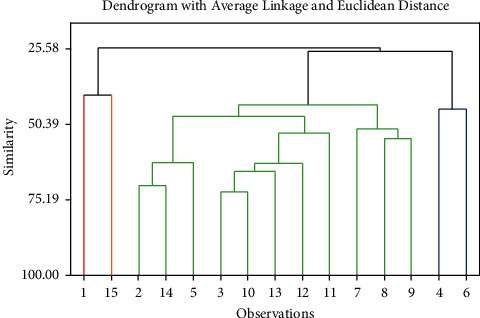
Dendrogram of cassava clones based on plant height in three months, fresh tuber yield in 10 months, starch content, starch yield, intensity of mite attack, harvest index, tuber diameter, and number of tubers/plant.

Dendogram analysis of 15 cassava clones based on plant height in three months, fresh tuber yield in 10 months, starch content, starch yield, intensity of mite attack, harvest index, tuber diameter, and number of tubers/plant is presented in Figure 1. If cutting is done with similarity of 40.47%, three clusters will be obtained (Table 3). There are 11 cassava clones (2; 14; 5; 3; 10; 13; 12; 11; 7; 8; 9) in the first cluster. The characteristics of these clones were low/medium tuber yield, low/medium starch yield, high/medium starch content, low/medium/high plant height, and susceptible/moderately resistant/resistant to mite. There are two clones (1 and 15) in second cluster. The characteristics of these clones were high tuber yield and starch yield, high starch content, low/medium plant height, and moderate resistance/resistance to mite. There are two clones (4; 6) in the third cluster. The characteristics of these clones were high tuber yield and starch yield, medium starch content, medium plant height, and moderate resistance/resistance to mite.

### 3.2. The Glass House Experiment

#### 3.2.1. Mite Population

**Figure 2:**
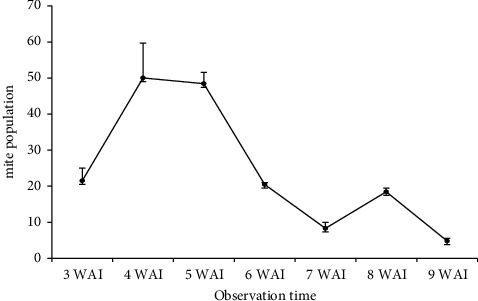
Total of mite population (±SE) at some observation times, glass house of ILETRI, 2018; WAI = weeks after infestation.

**Figure 3:**
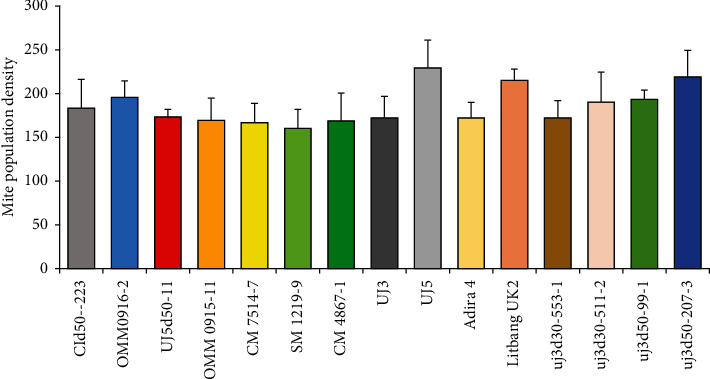
Total of mite population (±SE) of promising clones, glass house of ILETRI, 2018.

Figure 2 shows that mite population had increased in 3–4 weeks after infestation, and in 5 weeks after infestation, mite population had decreased. Mite population had increased in 8 weeks after infestation. Fluctuation of mite population depends on the condition of the host as feeds, place to take cover, and to breed. If condition of plant is fresh, mite population will increase. If quality of the host decreases, mite population will decrease. Mite population of tested clones had similar pattern.  Figure 3 shows that UJ5 had the highest mite population, 228 mites/plant, while SM 1219-9 had low mite population, 160 mites/plant. Insect population has a tendency to fluctuate as effect of environment factor. Environmental factors determine the increasing or decreasing of the mite population.

#### 3.2.2. The Damage Intensity of the Mite Attack

In glass house, yellowish spot along leaf veins was the first symptom of mite attack. This symptom can be seen in 2 weeks after infestation. After that, symptoms spread to form necrotic zones, so that color of leaf becomes brownish yellow. On the field, mite attack was started on leaf of lower part of plant and then attack leaf of upper part of plant. If population of mite is high, the color of the attacked leaf becomes brown and dry; then the leaf falls out.

In 2 weeks after infestation, the damage intensity of the mite attack was low 8–14% with average 11.39%; there were no differences between clones in response to mite attack. In 3 weeks after infestation, the damage intensity of the mite attack increased 17–36% with average 29%. Clone OMM 0915-11 had low damage intensity, similar to UJ5d50-11 (23.40%) and CM 4867-1 (22.66%) (Table 4).

In 4 weeks after infestation, the damage intensity reached 53%. In 5 weeks after infestation, clone UJ5d50-11 had the highest the damage intensity (69%), while clone OMM 0915-11 had the lowest damage intensity (55%), and significantly difference from the other clones. In 6 weeks after infestation, the damage intensity increased with the highest value 73%, and clone OMM 0915-11 had lowest value 59%. In 7 weeks after infestation, the damage intensity of nine clone (CId50-223, UJ5d50-11, CM 7514-7, UJ3, UJ5, UJ3d30-553-1, UJ3d30-511-2, UJ5d50-99-1, and UJ5d50-207-3) decreased because most of the leaves of those clones had fallen out. It means that those clones were more susceptible than the other clones. In 8 weeks after infestation, most clones had decreased intensity except clone OMM 0915-11, there was no fall-out leaf and dry leaf on this clone (Table 4).

In 6 weeks after infestation, there was no fall-out leaf on all clones tested, so this time was a good time to determine the response of clones to mite attack. During that time, OMM 0915-11 had low damage intensity (59%), and clone OMM0916-2, CM 4867-1, and Adira 4 had similar response to mite attack with this clone. Clone OMM0916-2, CM 4867-1, and Adira 4 were resistant, while UJ5d50-11, CM 7514-7, SM 1219-9, UJ3, UJ5, Litbang UK2, UJ3d30-553-1, UJ3d30-511-2, UJ5d50-99-1, and UJ5d50-207-3 were moderately resistant. Clone CId50-223 was susceptible (Table 5).

The interest of insects in plants is a response of stimulation that comes from plants, so insects come and eat the plant. There were five stages to find the host that was suitable for feeds, to take cover, and to breed. The finding of the host stage was affected by the light, temperature, humidity, wind, and the gravity. On finding the host stage, insects with a sense of sight and smell can find the right host, because insects were interested in stimulation from plant in the form of color, size, and shape of plant. After finding the host, stimulation of plant in the form of short distance encourages insects to stay on the plant. With the sense of touch and taste, insect will test the plant whether the plant can be accepted as the host or not. When the plant is suitable for the host, then insects will continue to eat. Table 4 shows that there were five clones with dark green leaf color and ten clones with green leaf color. There were four clones with dense pubescence, two clones with rare pubescence, and 9 clones with no pubescence on young leaf. Clones tested had 5-7 lobes, and most clones had a lanceolate lobe. The resistance clones (OMM0916-2, CM 4867-1, and OMM 0915-11) had a lanceolate lobe, a green leaf with no pubescence, so leaves become a little rough when touched (Table 6). This condition is not preferred by or interesting to mite as a place for eating, so mite attack on resistant clone was lower than the susceptible clone.

## 4. Discussion

The fresh tuber yield of OMM 0915-11 was the highest of 55.67 t/ha, significantly higher (58%–74%) than the existing varieties UJ3, UJ5, and Adira 4. This increasing value was equal to Rp 20.4 million to 23.6 million/ha or around US $ 1360–1576, if US $ 1 is equal to Rp 15,000 (Indonesian Rupiah). The assumption of the cost of the fresh tuber was Rp 1000/kg. On the other hand, this clone was resistant variety or highly resistant variety based on both the green house and field experiments. This clone is suitable for the area where the mite is a serious problem. The fresh tuber yields of mutant UJ5d50-99-1 and UJ5d50-207-3 were significantly higher than the original variety of them, i.e., UJ5. That means, there is an opportunity to increase the fresh tuber yields of cassava through mutation. On the other hand, this is in line with the research result reported by Sholihin and Mejaya [7]. This promising clone needed to be tested in some environments to evaluate the adaptability of the promising clone [8–11]. There was interaction between environment and genotype for the fresh tuber yield [12–14]. Cassava environment in Indonesia is very diverse, by which growth environment involved air temperature, kind of soil, type climate, rain fall, humidity, and so on.

The starch yield is multiplication between the fresh tuber yield and starch content. The highest starch yield (10.89 t/ha) had been achieved by clone UJ5d50-207-3. The mutant clone with original variety is UJ5. So there is an opportunity to increase the starch yield through mutation breeding. This is in line with the research result reported by Sholihin and Mejaya [7]. Clone UJ5d50-207-3 was moderately resistant to mite based on the field experiment and the green house experiment. So this clone is prospectively developed in area where the mite attack was not a serious problem such as the area with wet climate, for example, Lampung. Lampung is a center of cassava production in Indonesia and most cassava are used as row material for starch factory.

Clone OMM 0915-11 was resistant to mite based on the field experiment and the green house experiment. This clone can be used as parent in cassava hybridization as source gene for high yield and mite resistance. Clone OMM 0915-11 was also in different group with release variety such as UJ3, UJ5, Litbang UK2. This means that if clone OMM 0915-11 was used as a parent in crossing block to improve the released variety, there will be high opportunity to get a good progeny. This variety can be used as source of gene for resistance to mite. The different response of clones tested to the mite attack is predicted because of presence of antibiosis mechanism that inhibits the growth and development of insect. Antibiosis can decrease the insect population, reproductive power, period of reproduction, and death of nymph. This is in line with opinion of Astuti et al. [15] that resistance of plant to insect was because of existence of antibiosis that produced toxin that could kill insect or inhibit the growth of the insect. Antibiosis can decrease the insect population by decreasing the reproductive power and time of reproduction and increasing the death of nymph. On the other hand, Iswanto et al. [16] argue that there was a tolerant genotype related to the insect resistance, which was genotype that could decrease the loss of yield. Mariscal et al. [5] reported that additive and nonadditive gene effects play a role in the expression of cassava green mite density and cassava green mite leaf damage. This in line with what was reported by Wolfe et al. [17]. Ezenwaka et al. [18] reported that 35 single-nucleotide polymorphisms (SNPs) were significantly associated with cassava green mite severity, leaf pubescence, leaf retention, stay green, shoot tip compactness, and shoot tip size. They were possibly an indication of pleiotropy or the presence of closely linked genes that regulate their traits.

Evaluation of cassava clone to mite attack can be done through the glass house evaluation and the field evaluation. The disadvantage of the field evaluation is difficulty in the uniformity of mite attack, while the advantage of the field evaluation is that mite attack is similar to the real condition. According to Rahmathulla et al. [19], the mite population had a tendency to fluctuate as the effect of environment factor. The degree of influence of environment factor determines the insect population. The advantage of the green house evaluation is that uniformity of mite attack can be pursued by infestation, while disadvantage of the green house evaluation is that mite attack is not similar to the riel condition. Time for observation of the damage intensity of the mite attack is important in the selection and evaluation of cassava clone to develop a new variety for resistance to mite. Based on the green house evaluation, 6 weeks after infestation was a good time to determine the response of clones to mite attack. At that time, there was no fall-out leaf on all clones tested. Sholihin [20] reported that the time for observation of the damage intensity of the mite attack was 7 weeks after infestation. The philosophy in determining the time for observation of the damage intensity of the mite attack is the best time to distinguish the tested clone for resistance to mite. There is another method in selection and evaluation of cassava clone for mite resistance, that is, Markers Assisted Selection (MAS). Ezenwaka et al. [4] reported that there were nine candidate genes that related to cassava green mite resistance.

## 5. Conclusion

The average of tuber yield of OMM 0915-11 was the highest of 55.67 t/ha, significantly higher than the existing variety UJ3, UJ5, and Adira 4. This promising clone need to be tested in some different environments such as different kinds of soil, kinds of climate, and altitudes to know the adaptability of the promising clone. Clone OMM 0915-11 was resistant to mite based on both the field and the green house experiments. This clone has a chance to be released as new variety for high yield and resistance to mite. This clone also can be used as parent in cassava hybridization as source gene for high yield and mite resistance. The highest starch yield (10.89 t/ha) had been achieved by clone UJ5d50-207-3. Clone UJ5d50-207-3 was moderately resistant to mite based on both the field and the glass house experiments. So, both clones are prospective to be released as new variety for specific area such as the area with wet climate; one of them is in Lampung Province.

## Figures and Tables

**Table 1:** Plant height (cm ± SE), fresh tuber yield (*t* ± SE), starch content (±SE), and starch yield (*t* ± SE) of cassava promising clones in Malang, 2018.

No.	Clones	Plant height in 3 months (cm)	Fresh tuber yield (t/ha)	Starch content (%)	Starch yield (t/ha)
1	CM 3306-19	119 ± 8.85e	43.87 ± 4.95a–d	21.10 ± 0.58a	9.237 ± 0.90a–d
2	OMM0916-2	132 ± 8.54b–e	41.83 ± 2.92b–e	19.40 ± 0.28abc	8.150 ± 0.65b–e
3	OMM 0940-2	148 ± 11.43ab	30.47 ± 3.10e	19.20 ± 0.73abc	5.870 ± 0.63e
4	OMM 0915-11	120 ± 3.35e	55.67 ± 5.84a	16.83 ± 0.70c	9.307 ± 0.62abc
5	CM 7514-7	132 ± 11.80b–e	36.23 ± 1.94cde	19.07 ± 1.78abc	6.937 ± 0.77cde
6	SM 1219-9	126 ± 15.51cde	53.37 ± 2.93ab	19.33 ± 1.41abc	10.29 ± 0.73ab
7	CM 4867-1	125 ± 16.01cde	30.33 ± 5.18e	19.47 ± 1.64abc	5.927 ± 1.14e
8	UJ3^*∗*^	145 ± 5.50abc	35.27 ± 5.40de	19.80 ± 1.25abc	7.123 ± 1.53cde
9	UJ5^*∗*^	128 ± 2.31b–e	32.03 ± 3.18de	20.93 ± 0.78ab	6.687 ± 0.50cde
10	Adira 4^*∗*^	146 ± 4.81abc	35.10 ± 3.85de	18.47 ± 0.97abc	6.583 ± 1.04de
11	Litbang UK2^*∗*^	146 ± 2.89abc	48.10 ± 2.62abc	18.30 ± 1.41abc	8.817 ± 0.68a–d
12	UJ3d30-553-1	157 ± 4.87a	42.27 ± 7.31b-e	19.33 ± 1.15abc	8.023 ± 0.92b-e
13	UJ3d30-511-2	143 ± 3.30a–d	40.43 ± 1.77cde	17.87 ± 1.09bc	7.223 ± 0.43cde
14	UJ5d50-99-1	122 ± 10.58de	42.13 ± 3.38b–e	20.07 ± 0.74ab	8.517 ± 0.92a–e
15	UJ5d50-207-3	127 ± 9.08b–e	53.00 ± 6.96ab	20.53 ± 0.85ab	10.89 ± 1.55a
Mean		134	41.34	19.313	7.972
LSD 5%		21.2	12.35	3.133	2.698
C.V (%)		9	17	9	20

*Note*. ^*∗*^Varieties.

**Table 2:** Intensity of mite attack (±SE), harvest index (±SE), tuber diameter (cm ± SE), number of tubers/plant (±SE), and tuber length (cm ± SE) of cassava promising clones in Malang, 2018.

No.	Clone/variety	Intensity of mite attack (%)	Resistance category^*∗∗*^	Harvest index	Tuber diameter (cm)	Number of tubers/plant	Tuber length (cm)
1	CM 3306-19	51.32 ± 4.22	*S*	0.65 ± 0.04a	5.62 ± 0.43ab	16 ± 1.67	23 ± 1.02
2	OMM0916-2	33.32 ± 6.61	MR	0.55 ± 0.09bcd	4.46 ± 0.05d	18 ± 0.58	21 ± 2.34
3	OMM 0940-2	29.10 ± 1.06	*R*	0.54 ± 0.03bcd	4.43 ± 0.24d	13 ± 3.18	24 ± 0.65
4	OMM 0915-11	24.14 ± 8.26	*R*	0.49 ± 0.07cd	5.67 ± 0.27ab	16 ± 2.52	28 ± 2.67
5	CM 7514-7	44.19 ± 3.38	*S*	0.58 ± 0.05abc	4.43 ± 0.34d	16 ± 2.52	20 ± 0.35
6	SM 1219-9	35.24 ± 10.76	MR	0.54 ± 0.03bcd	4.59 ± 0.49cd	16 ± 0.67	29 ± 3.82
7	CM 4867-1	40.18 ± 5.11	MR	0.51 ± 0.05cd	4.97 ± 0.51a–d	14 ± 1.15	26 ± 4.42
8	UJ3^*∗*^	35.79 ± 1.26	MR	0.48 ± 0.04d	5.08 ± 0.87a–d	17 ± 2.40	20 ± 1.33
9	UJ5^*∗*^	47.50 ± 1.19	*S*	0.49 ± 0.04cd	5.87 ± 0.42a	15 ± 2.19	21 ± 1.85
10	Adira 4^*∗*^	32.84 ± 4.52	MR	0.52 ± 0.07cd	4.36 ± 0.40d	11 ± 0.88	21 ± 0.91
11	Litbang UK2^*∗*^	30.85 ± 8.75	MR	0.55 ± 0.05bcd	5.45 ± 0.51abc	14 ± 3.48	24 ± 2.37
12	UJ3d30-553-1	34.18 ± 1.38	MR	0.57 ± 0.05abc	4.42 ± 0.19d	14 ± 0.88	24 ± 1.01
13	UJ3d30-511-2	39.40 ± 0.38	MR	0.51 ± 0.02cd	4.48 ± 0.29d	14 ± 2.33	23 ± 2.00
14	UJ5d50-99-1	32.82 ± 3.71	MR	0.62 ± 0.03ab	4.61 ± 0.63cd	17 ± 1.73	23 ± 1.17
15	UJ5d50-207-3	41.37 ± 5.60	MR	0.57 ± 0.07abcd	4.74 ± 0.03bcd	21 ± 0.58	21 ± 2.31
Mean		36.82		0.55	4.88	16	23
LSD 5%		ns		0.092	0.951	ns	ns
CV (%)		26		11	12	23	17

*Note*. ^*∗*^Varieties; ^*∗∗*^*R* = resistant, MR = moderately resistant, and *S* = susceptible.

**Table 3:** Grouping of cassava clones based on plant height in three months, fresh tuber yield in 10 months, starch content, starch yield, intensity of mite attack, harvest index, tuber diameter, and number of tubers/plant.

Group	Cassava clones	Characteristics of group
1	2; 14; 5; 3; 10; 13; 12; 11; 7; 8; 9;	Low/medium tuber yield, low/medium starch yield, high/medium starch content, low/medium/high plant height, susceptible/moderately resistant/resistant to mite
2	1; 15	High tuber yield and starch yield, high starch content, low/medium plant height, susceptible/moderately resistant to mite
3	4; 6	High tuber yield and starch yield, medium starch content, medium plant height, moderately resistant/resistant to mite

**Table 4:** The damage intensity of mite attack (±SE) of promising clones, glass house, 2018.

Clones	Intensity of the mite attack (%) at							
	2 WAI^*∗∗*^	3 WAI	4 WAI	5 WAI	6 WAI	7 WAI	8 WAI	9 WAI
CId50-223	8.90 ± 1.07	29.81 ± 4.25abc	52.45 ± 1.51	64.56 ± 1.59ab	73.14 ± 1.49a	67.92 ± 8.75	60.17 ± 5.52abcd	42.19 ± 10.44
OMM0916-2	12.62 ± 1.23	29.51 ± 1.75abc	53.53 ± 2.81	62.12 ± 0.30b	63.53 ± 1.94bc	64.61 ± 5.99	59.19 ± 4.38abcd	51.89 ± 3.24
UJ5d50-11	11.14 ± 1.08	23.40 ± 3.54cd	50.63 ± 3.96	68.83 ± 1.56a	70.51 ± 1.97a	56.56 ± 13.41	47.35 ± 11.60cd	37.78 ± 6.24
OMM 0915-11	9.62 ± 1.07	17.17 ± 2.78d	39.06 ± 11.23	55.58 ± 4.58c	59.07 ± 1.34c	62.75 ± 0.96	70.01 ± 3.14a	66.92 ± 3.65
CM 7514-7	12.32 ± 0.66	34.71 ± 4.50ab	57.48 ± 1.27	64.95 ± 0.91ab	68.66 ± 0.51ab	65.66 ± 0.70	52.94 ± 3.58abcd	44.00 ± 2.57
SM 1219-9	11.55 ± 0.83	26.21 ± 1.93abcd	65.23 ± 13.57	63.28 ± 2.32ab	68.08 ± 1.72ab	71.87 ± 4.15	59.31 ± 10.13abcd	53.52 ± 10.99
CM 4867-1	10.44 ± 1.14	23.66 ± 4.13cd	45.87 ± 4.59	61.14 ± 0.22bc	63.95 ± 1.84bc	69.06 ± 2.30	61.50 ± 4.91abcd	45.55 ± 15.02
UJ3^*∗*^	10.27 ± 1.20	27.64 ± 4.05abc	46.00 ± 4.47	64.15 ± 2.47ab	70.19 ± 4.36 a	54.57 ± 11.72	42.52 ± 12.08d	39.77 ± 12.48
UJ5^*∗*^	13.18 ± 2.77	32,23 ± 4.42abc	56.53 ± 2.60	66.43 ± 1.92ab	67.26 ± 2.71ab	63.96 ± 4.76	50.70 ± 7.40abcd	46.59 ± 8.36
Adira 4^*∗*^	9.23 ± 1.33	31.87 ± 3.04abc	58.42 ± 0.78	62.09 ± 0.48b	63.27 ± 0.70bc	72.05 ± 2.35	67.55 ± 1.06ab	60.21 ± 1.44
Litbang UK2^*∗*^	10.90 ± 1.55	33.61 ± 3.20ab	54.45 ± 2.22	62.61 ± 1.62ab	70.45 ± 1.46a	74.26 ± 3.45	65.50 ± 6.09abc	58.41 ± 8.62
UJ3d30-553-1	14.61 ± 2.51	32.63 ± 6.39abc	51.83 ± 0.94	62.22 ± 2.30b	70.12 ± 3.36a	60.71 ± 5.20	49.34 ± 4.20bcd	40.71 ± 7.17
UJ3d30-511-2	10.52 ± 0.06	33.65 ± 4.04ab	56.34 ± 1.60	64.00 ± 3.11ab	67.28 ± 1.40ab	52.71 ± 5.15	46.87 ± 5.83cd	47.27 ± 5.95
UJ5d50-99-1	14.04 ± 1.24	36.03 ± 2.1 a	58.00 ± 0.80	65.25 ± 1.06ab	71.56 ± 1.79a	68.66 ± 5.90	47.86 ± 2.44bcd	41.1 ± 3.79
UJ5d50-207-3	11.54 ± 1.87	25.07 ± 5.67bcd	51.73 ± 3.96	64.22 ± 1.22ab	71.04 ± 1.87a	62.76 ± 4.82	55.17 ± 6.16abcd	52.00 ± 7.47
Average	11.39	29.08	53.17	63.43	67.81	64.60	55.73	48.53
Sd	1.69	5.31	6.28	2.92	3.78	6.51	8.35	8.47
LSD 5%	ns	8.633	ns	5.594	5.337	ns	16.87	ns
CV (%)	21.09	17.75	16.45	5.27	4.71	14.26	18.1	23.7

*Note*. ^*∗*^ = varieties; ^*∗∗*^WAI = weeks after infestation.

**Table 5:** The damage intensity of mite attack and susceptibility class of promising clones, glass house, 2018.

Clones	The damage intensity of the mite attack in 6 WAI (%)	Susceptibility class^*∗∗*^
CId50-223	72.14	*S*
OMM0916-2	63.53	*R*
UJ5d50-11	70.51	MR
OMM 0915-11	59.07	HR
CM 7514-7	68.66	MR
SM 1219-9	68.08	MR
CM 4867-1	63.95	*R*
UJ3^*∗*^	70.19	MR
UJ5^*∗*^	67.26	MR
Adira 4^*∗*^	63.27	*R*
Litbang UK2^*∗*^	70.45	MR
UJ3d30-553-1	70.12	MR
UJ3d30-511-2	67.28	MR
UJ5d50-99-1	71.56	MR
UJ5d50-207-3	71.04	MR

*Note*. ^*∗*^Varieties; ^*∗∗*^HR = highly resistant; *R* = resistant; MR = moderately resistant; *S* = susceptible.

**Table 6:** Character of leaf morphology and susceptibility class of promising clones, green house of ILETRI, 2018.

Clones	Character of leaf morphology of promising clones				
	Mature leaf color	Pubescence	Number of lobes	Shape of lobe	Susceptibility class
CId50-223	Green	Absent	5	Obovate-lanceolate	*S*
OMM0916-2	Green	Absent	5	Lanceolate	*R*
UJ5d50-11	Dark green	Absent	7	Lanceolate	MR
OMM 0915-11	Green	Absent	7	Lanceolate	HR
CM 7514-7	Green	Present/dense	7	Lanceolate	MR
SM 1219-9	Green	Present/rarely	7	Lanceolate	MR
CM 4867-1	Green	Present/rarely	7	Lanceolate	*R*
UJ3^*∗*^	Dark green	Present/dense	5	Lanceolate	MR
UJ5^*∗*^	Green	Absent	5	Lanceolate	MR
Adira 4^*∗*^	Dark green	Absent	6	Obovate-lanceolate	*R*
Litbang UK2^*∗*^	Green	Absent	7	Lanceolate	MR
UJ3d30-553-1	Dark green	Present/dense	5	Lanceolate	MR
UJ3d30-511-2	Dark green	Present/dense	5	Lanceolate	MR
UJ5d50-99-1	Green	Absent	5	Lanceolate	MR
UJ5d50-207-3	Green	Absent	7	Obovate-lanceolate	MR

*Note*. ^*∗*^Varieties; ^*∗∗*^HR = highly resistant; *R* = resistant; MR = moderately resistant; *S* = susceptible.

## Data Availability

The data used to support the findings of this study are available from the corresponding author upon request.
